# Two *fatty acyl reductases* involved in moth pheromone biosynthesis

**DOI:** 10.1038/srep29927

**Published:** 2016-07-18

**Authors:** Binu Antony, Bao-Jian Ding, Ken’Ichi Moto, Saleh A. Aldosari, Abdulrahman S. Aldawood

**Affiliations:** 1 King Saud University, Department of Plant Protection, Chair of Date Palm Research, College of Food and Agricultural Sciences, Riyadh 11451, Saudi Arabia; 2 Lund University, Department of Biology, Lund 22362, Sweden; 3 RIKEN, Lipid Biology Laboratory, Wako, Saitama 351-0198, Japan

## Abstract

Fatty acyl reductases (FARs) constitute an evolutionarily conserved gene family found in all kingdoms of life. Members of the FAR gene family play diverse roles, including seed oil synthesis, insect pheromone biosynthesis, and mammalian wax biosynthesis. In insects, FAR genes dedicated to sex pheromone biosynthesis (pheromone-gland-specific fatty acyl reductase, pgFAR) form a unique clade that exhibits substantial modifications in gene structure and possesses unique specificity and selectivity for fatty acyl substrates. Highly selective and semi-selective ‘single pgFARs’ produce single and multicomponent pheromone signals in bombycid, pyralid, yponomeutid and noctuid moths. An intriguing question is how a ‘single reductase’ can direct the synthesis of several fatty alcohols of various chain lengths and isomeric forms. Here, we report two active pgFARs in the pheromone gland of *Spodoptera*, namely a semi-selective, C14:acyl-specific pgFAR and a highly selective, C16:acyl-specific pgFAR, and demonstrate that these pgFARs play a pivotal role in the formation of species-specific signals, a finding that is strongly supported by functional gene expression data. The study envisages a new area of research for disclosing evolutionary changes associated with C_14_- and C_16_-specific FARs in moth pheromone biosynthesis.

Insects use sophisticated chemical communication systems, including behavior-modifying pheromones, and these systems reinforce intra- and interspecific reproductive isolation and thus play pivotal roles in the evolutionary process. The insect order Lepidoptera (moth and butterflies) contains the second largest number of species (approximately 180,000 species described to date), and their richness and diversity are linked to pheromone-based communication and sophisticated chemosensory systems^1^,^2^,^3^. In moths, chemical communication is the major factor in the premating isolation mechanism, and sex pheromone differences inhibit successful mating and disfavor reproductive success^4^. The female signal and male response are highly species specific, but the mechanism regulating concomitant changes in signals and responses has not been established. These changes must occur at the gene level, and it is important to identify and characterize the genes responsible for pheromone production and to thus provide conclusive proof regarding pheromone-driven evolution in Lepidoptera^5^,^6^. The pheromone race is generally described in moths, and the variability in chemical mating signals and responses is genetically determined and under stabilizing selection^7^,^8^,^9^. Studies over the last two decades have pinpointed that the epigenetic effect of pheromone-driven adaptive evolution is one of the major factors driving the successful diversification of Lepidopteran insects^10^. In moths, a few substitutions in critical amino acids in the key pheromone biosynthetic enzymes are sufficient to create a novel pheromone component^11^,^12^. Few studies have reported the genetic basis of pheromone divergence in insects due to a lack of information on the key pheromone biosynthetic enzymes and the absence of a model insect species.

Moth pheromone compounds are derived from saturated C_10_-C_18_ fatty acyl moieties that are modified in the pheromone gland (PG) through the addition of functional groups, such as alcohol, acetate ester, or aldehyde. The biosynthesis of pheromone bouquets involves specialized enzymes, including fatty acyl-CoA desaturases, fatty acyl-CoA reductases (FARs), alcohol oxidases and fatty alcohol acetyltransferases^13^. One of the key enzymes involved in the production of oxygenated functional groups is FAR, which catalyzes the reduction of fatty acyl-CoA precursors to the corresponding alcohols^13^. Many of the pgFARs reported to date are selective regarding the carbon chain length of the substrate and show reduced activity to substrate chains that are shorter or longer than those of the original precursors^7^,^11^,^14^,^15^,^16^,^17^,^18^. The current information reveals a single pgFAR that acts on several C_14_ or C_16_ acyls^11^,^14^,^16^,^17^,^19^. However, several moths, including *Spodoptera* species, utilize multicomponent C_12_, C_14_ and C_16_ alcohol and acetate ester pheromone blends. Of the thirty *Spodoptera* species, the pheromone mixtures identified from 18 species (see Table S1) are composed of a multicomponent blend of isomerically related mono- and di-unsaturated C_12_, C_14_, and C_16_ fatty alcohols and acetates^20^,^21^,^22^. In the PG, an intriguing question is how a single reductase enzyme system can act on several fatty acyls of various chain lengths and isomeric forms. The present study describes an extensive search of the *Spodoptera exigua* PG that revealed two active pgFAR candidates, and a heterologous gene expression study in yeast demonstrated that each FAR plays a specific role in the reduction step. The two pgFARs belong to a unique pgFAR subfamily of Lepidoptera and are expressed solely in the PG of *S. exigua*. One enzyme exhibits broad selectivity toward C_14_ fatty acyl compounds, and the other has high specificity toward C_16_ fatty acyl compounds. Our study provides the first description of the involvement of multiple pgFARs with different selectivities and preferences for C_14_ and C_16_ fatty acyl moieties in pheromone biosynthesis in moths.

## Results

### Cloning of FAR candidates

**Figure 1:**
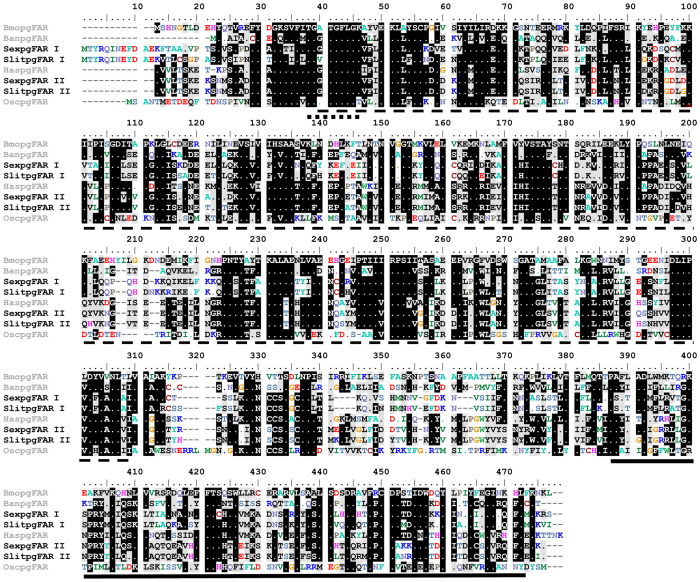
Protein sequence alignment of SexpgFAR I and SexpgFAR II with *B. mori* (BmopgFAR) (GenBank accession no. BAC79426), *O. scapularis* (OscpgFAR) (ACJ06520) *B. anynana* (BanpgFAR) (AGD98719) and *H. assulta* (HaspgFAR) (JF709977). The sequence alignments were computed in Clustal W and edited in BioEdit (v.7.2.5). The amino acids shaded in black or grey indicate identical residues or conserved substitutions. The FAR protein structure includes an N-terminal Rossmann fold (NAD(P)(+)-binding domain) (dashed line), the NADPH-binding motif (dotted line), and the sterile domain (thick dark line).

In the search for pgFARs, we identified eight FAR-like genes (FAR-I to FAR-VIII, GenBank Accession Nos. KF805977-KF805983 and KR781119-KR781121) based on their deduced amino acid sequences, which contain a conserved NAD(P)H-binding motif 23,24,25. BLASTx searches of the non-redundant (*nr*) protein database identified two candidate FARs from *S. exigua* that show significant identity with those of the nymphalid butterfly *Bicyclus anynana* (AGD98718) [50% amino acid (aa) identity and 67% similarity]^26^ and the noctuid moth *Helicoverpa assulta* (45% identity and 65% similarity)^17^. We named the candidates SexpgFAR I and SexpgFAR II. The full-length SexpgFAR I and SexpgFAR II cDNA transcripts contain 1,616 bp and 1,526 bp, respectively, and encompass open reading frames (ORFs) of 1,377 base pair (bp) and 1,362 bp, corresponding to proteins of 459 aa (SexpgFAR I) and 454 aa (SexpgFAR II) with predicted molecular weights of 51.7 and 51.4 kDa, respectively. SexpgFAR I and SexpgFAR II share 39% and 42% identity with the *B. mori* pgFAR^16^, 33% and 34% identity with the human FAR^27^, and 27% and 25% identity with the Jojoba FAR^28^, respectively (Table 1). The nucleotide and aa comparison of SexpgFAR I and SexpgFAR II with other moth pgFARs is provided in Table 1. SexpgFAR I and SexpgFAR II share 44% aa identity (<65% similarity) and more than 50% nucleotide identity (62% similarity) and possess highly conserved NAD(P)H-binding Rossmann fold domains (Fig. 1). The pgFARs contain a GXXGXX(G/A) motif at their N terminus that resembles the canonical ADP-binding domain, and this motif is likely involved in binding to NAD(P)H; these proteins also contain a C-terminal sterile domain and an epimerase domain (Fig. 1)^23^,^24^,^25^. Both pgFARs contain the classic YXXXK active-site motif of the short-chain dehydrogenase/reductase superfamily and are members of the short-chain dehydrogenase/reductase (SDR) family^29^. The results of a sequence analysis using the GenBank database identified both pgFARs as alcohol-forming long-chain FARs (E.C.1.2.1.50). The endoplasmic reticulum localization of SexpgFAR I and SexpgFAR II, which is a typical characteristic of pgFAR proteins, was identified using the Euk-mPloc 2.0 server^30^. We also cloned the pgFARs from *S. littoralis* (hereafter named SlitpgFAR I and SlitpgFAR II; GenBank Accession Nos. KR781119 and KR781120) by RACE-PCR cDNA amplification and subsequent Sanger sequencing. These pgFARs encompass ORFs of 1,438 and 1,362 bp, corresponding to proteins of 476 and 454 aa, respectively. SlitpgFAR I and SexpgFAR I share 84% aa identity, SlitpgFAR II and SexpgFAR II share 93% aa identity, and SlitpgFAR I and SlitpgFAR II share 44% aa identity. A pair-wise sequence comparison of SexpgFAR I and SlitpgFAR I with other noctuid pgFARs (*Heliothis* and *Helicoverpa* spp.) showed more than 44% aa identity, whereas a comparison of SexpgFAR II and SlitpgFAR II with the same FARs (*Heliothis* and *Helicoverpa* spp.) showed 73% aa identity (Table 1).

### Tissue specificity studies and quantitative PCR identified a pheromone gland-specific FAR

**Figure 2:**
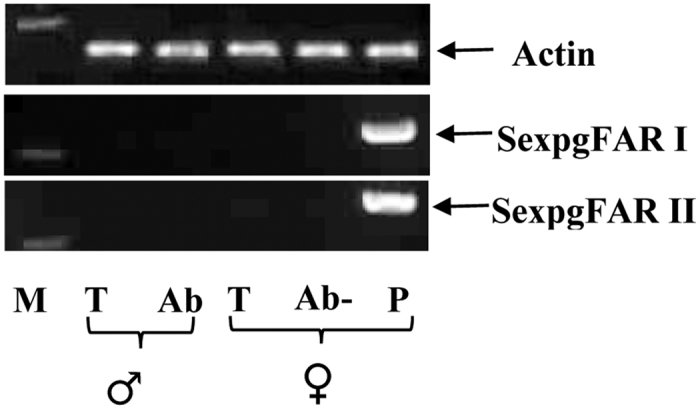
Tissue-specific expression of SexpgFAR I and SexpgFAR II by RT-PCR. Amplicon sizes: SexpgFAR I, 262 bp; SexpgFAR II, 286 bp and actin, 217 bp. The 250 bp DNA marker (M) is shown. T: thorax; Ab: abdomen; P: pheromone gland; Ab-: abdomen without PG.

Our RT-PCR results of the pgFAR gene expression pattern in different tissues from male and female *S. exigua* moths revealed that both SexpgFAR I and SexpgFAR II were expressed only in females and were exclusively expressed in the PG (Fig. 2). The PG-specific expression of both FARs suggests that these proteins may play a selective role in pheromone biosynthesis^31^. In contrast, all other FARs (FARI-FARVI) were found to be broadly distributed in various tissues, and their expression was not female-specific (data not shown). We performed quantitative PCR (qPCR) using cDNA synthesized from 2- to 3-day-old female moths, and our data showed that transcript abundances of both SexpgFAR I and SexpgFAR II were the same in female moths, indicating that both pgFARs exhibit a similar gene expression pattern in the PG (Fig. S1).

### Phylogenetic analysis revealed that SexpgFAR I and SexpgFAR II are orthologous to Lepidopteran pgFARs

**Figure 3:**
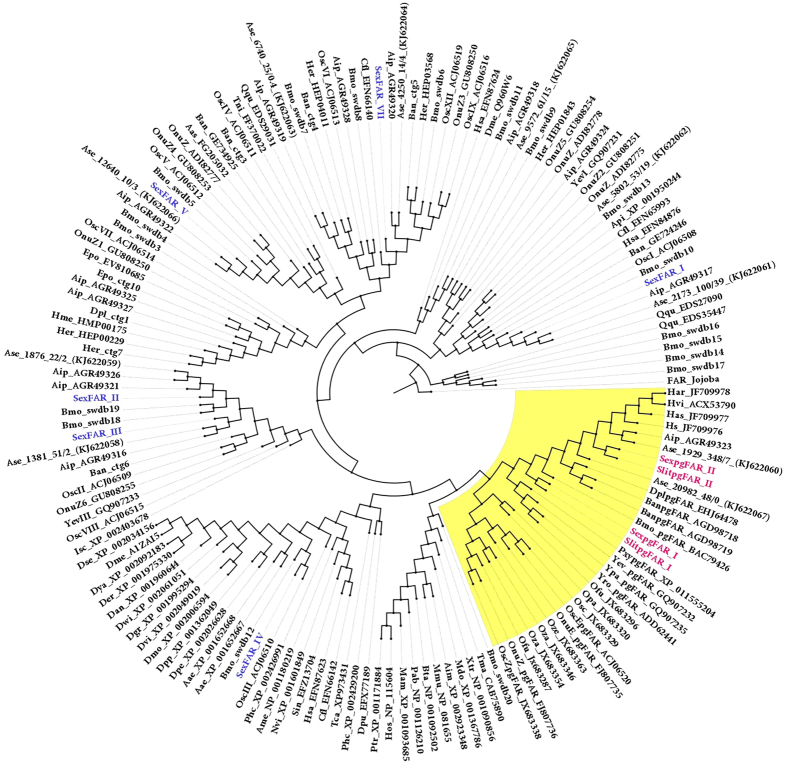
Maximum likelihood (ML) tree of the *fatty acyl reductase* proteins from *Lepidoptera*. The ML analysis was computed using MEGA (v.6.0)^49^. [The Jones-Taylor-Thornton (JTT) model for the ML heuristic search methods was the Nearest-Neighbor Interchange]. The Lepidopteran FAR-like sequences were retrieved from the GenBank, EST and TSA databases using BLASTx searches, and SexpgFAR I and SexpgFAR II were used as the query. The protein sequences were aligned using MUSCLE^48^. The branch containing Jojoba FAR^28^ was used as outgroup to root the tree. Other FAR-like proteins characterized from *S. exigua* are shown in blue. The pgFAR clade is highlighted in yellow. The GenBank accession numbers are indicated.

We constructed a phylogeny of Lepidopteran FARs using the FAR genes collected from the GenBank, genomic, transcriptomic and EST databases. Both pgFARs from *S. exigua* fall under the Lepidopteran pgFAR clade and were grouped with the pgFARs from *B. mori*^16^, *Ostrinia* spp.^7^,^11^,^14^, *Yponomeuta* spp.^19^, *Agrotis* spp.^15^,^32^ and *Heliothis* and *Helicoverpa* spp.^17^ (Fig. 3). Interestingly, SexpgFAR I formed a cluster with the *B. anynana* (BanpgFAR) and *B. mori* (BmopgFAR) pgFARs, which are reported to be C16-acyl-specific FARs^16^,^26^, and with the *Danaus plexippus* (DplpgFAR) and *Plutella xylostella* (PxypgFAR) pgFARs (Fig. 3), which are uncharacterized but predicted to be C16-acyl-specific FARs because the pheromone compounds are C16-acyl derivatives. SexpgFAR II was grouped with the noctuid pgFARs, which are known to be semi-selective C14 acyl-specific FARs (Fig. 3). In the gene tree, the *Spodoptera* pgFAR II was clustered in proximity to those pgFARs with broad specificity, whereas the *Ostrinia* pgFARs remain a separate group that is highly selective^11^. The orthologous SlitpgFAR I and SlitpgFAR II from *S. littoralis* were also grouped into the Lepidopteran pgFAR clade (Fig. 3).

### A yeast functional assay revealed that SexpgFAR I and SexpgFAR II prefer C_16_ and C_14_ fatty acyl substrates, respectively

**Figure 4:**
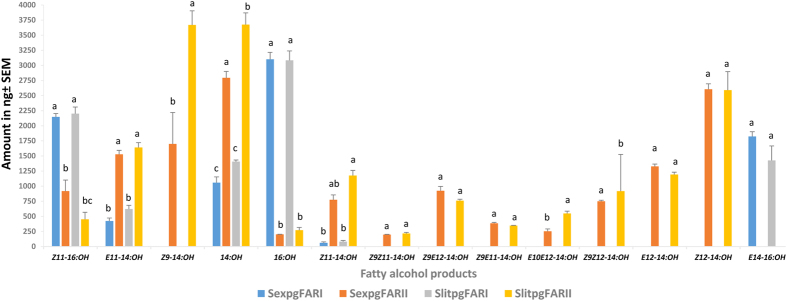
Analysis of the yeast construct functional assay showing the fatty alcohol production of SexpgFAR I and SexpgFAR II from *S. exigua* and SlitpgFAR I and SlitpgFAR II from *S. littoralis*. The recombinant yeast strain *S. cerevisiae* was supplemented with 0.5 mM concentrations of various fatty-acyl methyl ester (FAME) substrates for 48 h at 30 °C, and the *n*-hexane extracts of the yeast pellets were subjected to GC-MS analysis. The fatty alcohol production was calculated using a previously described method^11^. The bars represent the SEMs of fatty alcohol production (in nanograms) of 3 biological replicates. The bars with different letters within each series are significantly different (*P* < 0.05).

To address the functional role of the *S. exigua* pgFARs, we cloned the SexpgFAR I and SexpgFAR II ORFs into the pYES2.1 shuttle vector and transformed them into *InvSc1* yeast cells. We also established *InvSc1* yeast cells that were transformed with an empty vector (negative control) and *B. mori* pgFAR (positive control)^16^ to ensure that the production of alcohol in the yeast cells was due to expression of the recombinant pgFAR genes. We initially performed the functional assay with the SexpgFAR I- and SexpgFAR II-transformed yeast cells supplemented with 0.5 mM hexadecanoic acid methyl ester (C16:COOMe) and tetradecanoic acid methyl ester (C14:COOMe). Both pgFARs were found to be capable of reducing both the exogenous and endogenous C_14_ acyl and C_16_ acyl compounds that naturally occur in yeast (Fig. S2A). However, the SexpgFAR I-transformed yeast cells produced a significantly higher quantity of C_16_ fatty alcohol, whereas the SexpgFAR II-transformed yeast cells selectively reduced the amount of C_14_ fatty acids and produced significantly less C_16_ alcohol (Figs S2A and 4; Table 2). The SexpgFAR I-transformed yeast cells produced more than five fold greater amounts of 16:OH compared with the SexpgFAR II-transformed yeast cells, which is an even higher concentration of 16:OH than that produced by BmopgFAR (Fig. S2; Table 2). SexpgFAR II produced a significantly higher concentration of C14:OH (Figs S2A and 4). None of the corresponding C_14_ and C_16_ fatty alcohol compounds were observed in the negative control experiment (Fig. S2A–C). To determine whether both FARs reduce the (*Z*)-11-hexdecenoic acid (*Z*11-16:acyl), one of the minor pheromone precursors, we performed a functional assay with *Z*11-16:acyl, and both pgFAR-transformed yeast constructs produced *Z*11-16:OH. However, SexpgFAR I produced significantly more *Z*11-16:OH than SexpgFAR II (Figs S2B and 4). The SexpgFAR II-transformed yeast cells produced minor amounts of *Z*11-16:OH (6% of the corresponding production of saturated C_16_ alcohol), indicating reduced activity (Table 2). The majority of the unused *Z*11-16:COOMe from the yeast cells is retained in the hexane extract (Fig. S2B). We then tested a rare C_16_ pheromone precursor (*E*14-16:acyl, (*E*)-14-hexdecenoic acid), an intermediate pheromone precursor of the Asian corn borer (ACB) *O. furnacalis* that is not found in the glands of *Spodoptera* species (Table S1), to test the substrate selectivity of SexpgFAR I and determine whether it can reduce unsaturated C16-acyls other than *Z*11-16:acid. The SexpgFAR I-transformed yeast cells produced high levels of *E*14-16:OH, and these levels were even higher than those found with the positive control *B. mori* pgFAR (Figs S2C and 4). SexpgFAR II was unable to convert *E*14-16:COOMe to the corresponding alcohol (Fig. S2C). A summary of pgFAR activities based on the corresponding fatty alcohol derivatives produced from different fatty acyl substrates is provided in Table 2. These data suggest that *Spodoptera* pgFAR I has evolved the ability to reduce a broad set of C_16_ fatty acyls. We repeated the functional assay experiment with the SlitpgFAR I and SlitpgFAR II yeast constructs and obtained similar results (Fig. 4).

### SexpgFAR I is selective for *Z*11-16:acyl, and SexpgFAR II is involved in saturated and unsaturated C_14_ fatty acyl reduction

A series of yeast functional assay experiments was performed with different saturated and monounsaturated *Spodoptera* pheromone precursors. We supplemented the SexpgFAR I-transformed yeast cells with *Z*11-16:COOMe, (*E*)-11-tetradecenoic acid Me (*E*11-14:COOMe), (*Z*)-9-tetradecenoic acid Me (*Z*9-14:COOMe) and (*Z*)-11-tetradecenoic acid Me (*Z*11-14:COOMe) in separate experiments (single-substrate assay). Hexane extraction of the yeast pellets revealed large amounts of *Z*11-16:OH (Figs S2B and 4) and traces of *E*11-14:OH. None of the other substrates could be reduced to the corresponding alcohols (Fig. S3A–C). The supplementation of the SexpgFAR II-transformed yeast cells with the aforementioned compounds in single-substrate assay experiments converted all of the 14C fatty acid methyl ester (FAME) precursors into the corresponding alcohols (Fig. S3A–C). These studies demonstrated that SexpgFAR I is actively involved in the reduction of *Z*11-16:acyl (Fig. S2B) and that SexpgFAR II actively reduces all of the C_14_ mono fatty acyl moieties (Figs S2, S3A–C and 4). Similar functional assay experiments were performed with SlitpgFAR I and SlitpgFAR II, and these yielded similar results (Fig. 4).

To further test the selectivity of pgFAR II, we performed an additional functional assay with *E*- and *Z*12-14:acid, a rare pheromone precursor of ACB *O. furnacalis*^33^ that is not found in the PG of *Spodoptera* (Table S1). Interestingly, the SexpgFAR II-transformed yeast cells reduced (*E*)-12-tetradecenoic acid Me (*E*12-14:COOMe) and (*Z*)-12-tetradecenoic acid Me (*Z*12-14:COOMe) to the corresponding alcohols, and we observed significant *Z*12-14:OH production compared with all the other monounsaturated compounds (Figs 4 and S4). The results indicate the broad selectivity of pgFAR II toward C_14_ acyls. SexpgFAR I was unable to reduce the *E*- and *Z*12-14:acid compounds (Fig. S4). We repeated the same experiment with *B. mori* pgFAR, and, as expected, BmopgFAR was unable to reduce the *E*- and *Z*12-14:acids (data not shown), indicating the functional similarity of *S. exigua* pgFAR I and *B. mori* pgFAR. Both have evolved to reduce C_16_ acyl compounds.

We performed another set of experiments with di-unsaturated pheromone precursors. None of the corresponding fatty alcohol compounds were produced in the yeast SexpgFAR I functional assay (Fig. S3D–H). The SexpgFAR II-transformed yeast cells produced the corresponding alcohols from the di-unsaturated C_14_ fatty acid pheromone precursors (Fig. S3D–H). Similar results were obtained with SlitpgFAR I and SlitpgFAR II (Fig. 4).

### *Spodoptera* pgFAR II is selective for *Z*9-14:acid and *Z*9*E*12-14:acid precursors

**Figure 5:**
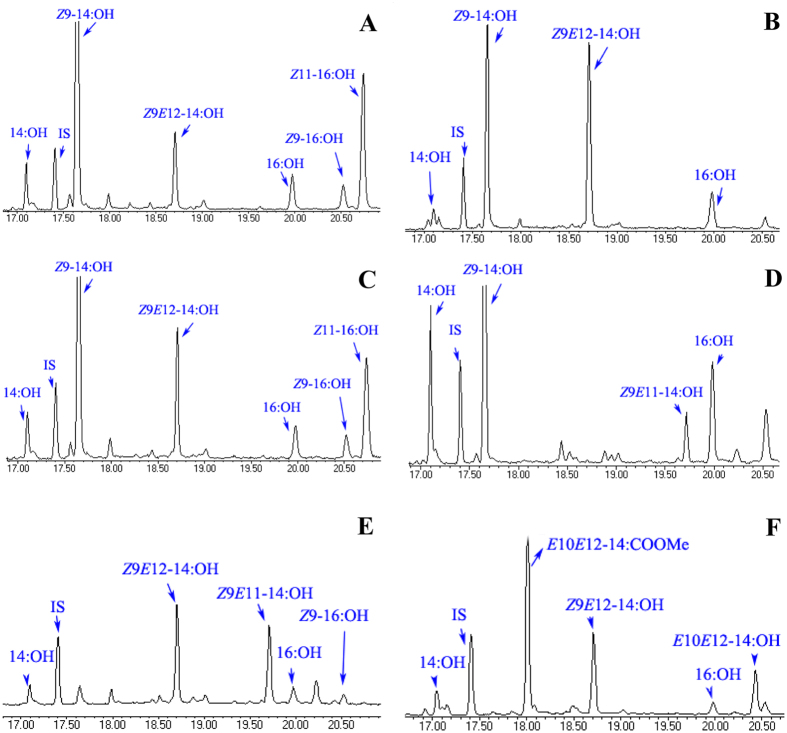
(**A–F**) Functional assay and GC-MS analysis of yeast (*InvSc1*) transformed with the SexpgFAR II construct and supplemented with different blends of 0.5 mM (total concentration) FAMEs: (**A**) *Z*9*E*12-14:COOMe, *Z*9-14:COOMe and *Z*11-16:COOMe (34:62:4), (**B**) *Z*9*E*12-14:COOMe and *Z*9-14:COOMe (34:62), and (**C**) *Z*9*E*12-14:COOMe, *Z*11-16:COOMe and *Z*9-14:COOMe (1:1), (**D**) *Z*9*E*11-14:COOMe and *Z*9-14:COOMe (1:1), (**E**) *Z*9*E*12-14:COOMe and *Z*9*E*11-14:COOMe (1:1) and (**F**) *Z*9*E*12-14:COOMe and *E*10*E*12-14:COOMe (1:1). *Spodoptera* pgFAR reduces the 14:acid, 16:acid and *Z*9-16:acid compounds that are naturally present in the yeast.

The hexane extract of the female *S. exigua* pheromone gland contained five major pheromone compounds^22^. However, the solid-phase microextraction (SPME) analysis revealed a mixture of (*Z,E*)-9,12-tetradecadienyl acetate (*Z*9*E*12-14:OAc), *Z*9-14:OAc, *Z*11-16:OAc and *Z*9-14:OH at a 34:40:4:22 ratio^22^. Thus, in the functional assay, we supplemented the SexpgFAR II-transformed yeast cells with the above-described mixture of three FAME compounds. Interestingly, we obtained 62% *Z*9-14:OH and 22% *Z*11-16:OH, but the yeast cells produced significantly less *Z*9*E*12-14:OH (16%) (Fig. 5A). We compared the production of *Z*11-16:OH with that observed in our previous single-substrate assay (see Fig. 4) and found that the same level of *Z*11-16:OH production by SexpgFAR II. Because we supplemented the yeast cells with higher quantities of *Z*11-16:COOMe (0.5 mM) in the single-substrate assays, SexpgFAR II, despite its low reductase activity to the *Z*11-16:acid, produced nearly half of the *Z*11-16:OH amount produced by SexpgFAR I (Fig. 4). The reason for this finding might be the availability of a high quantity of substrate [produced by elongation of the *Z*9-14:acid to the *Z*11-16:acid through endogenous yeast elongase activity^34^] to produce higher levels of *Z*11-16:OH in the *S. exigua* functional assay. However, in a yeast functional assay with an *S. exigua* blend, SexpgFAR II showed higher reductase activity toward the *Z*9-14:acid and produced the same amount of *Z*9-14:OH. These results indicate that SexpgFAR II is highly selective for the *Z*9-14:acid precursors and that SexpgFAR II activity might be highly restricted to C14 acyls *in vivo*. Alternatively, the introduction of the C16:acyl substrates (as in the case of the *Z*11-16:acid in our experiment) would reduce enzyme activity and decrease production of the di-unsaturated fatty alcohol (Fig. 5A). This experiment also supports the notion that SexpgFAR I is actively involved in *Z*11-16:OH synthesis in the PG. Additional functional assays were independently performed using ratios of *Z*9-14:COOMe to *Z*9*E*12-14:COOMe equal to 62:34 and 1:1. Both cases showed increased *Z*9-14:OH production (Fig. 5B), and we obtained fatty alcohol ratios of 52:48 and 74:26, respectively, which are slightly higher than the *Z*9-14:OH production level that was previously reported^22^. These results reiterate the selective nature of SexpgFAR II in systems supplemented with only C_14_ mono and di-unsaturated precursors. However, significantly higher amounts of *Z*9-14:OH were produced in the 1:1 functional assay (Fig. 5C). To further confirm the selectivity of SexpgFAR II toward *Z*9-14:acyls, we conducted another functional assay with a ratio of *Z*9-14:acyl to *Z*9*E*11-14:acyl (major pheromone precursor of *S. littoralis*)^21^ equal to 1:1 and obtained significantly higher *Z*9-14:OH production with a blend ratio of 73:27 (Fig. 5D). These experiments reiterate the fact that SexpgFAR II prefers the *Z*9-14:acid substrate.

To identify the second most preferred substrate of *S. exigua* pgFAR, we conducted two separate multi-substrate assay experiments, one with concentration ratios of *Z*9*E*12-14:COOMe to *Z*9*E*11-14:COOMe equal to 1:1 and another with concentration ratios of *Z*9*E*12-14:COOMe and *E*10*E*12-14:COOMe (pheromone component of *S. littoralis*^21^) equal to 1.1. In both experiments, we obtained higher *Z*9*E*12-14:OH ratios, indicating that the *Z*9*E*12-14:acid is the second most preferred precursor for SexpgFAR II (Fig. 5E,F). Taken together, these results demonstrate that SexpgFAR II exhibits specificity for *Z*9-14:acid and *Z*9*E*12-14:acid precursors.

**Figure 6:**
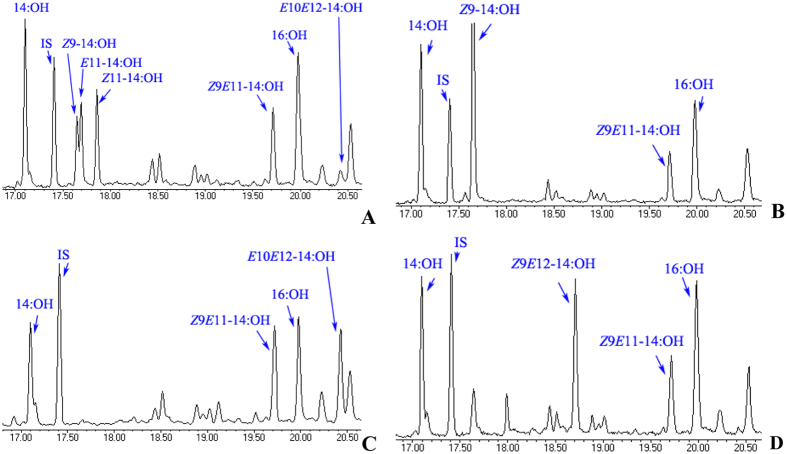
(**A–D**) Functional assay and GC-MS analysis of yeast (*InvSc1*) transformed with the SlitpgFAR II construct and supplemented with different ratios of 0.5 mM FAMEs: (**A**) *Z*9*E*11-14:COOMe, *Z*9-14:COOMe, *E*11-14:COOMe, 14:COOMe, *Z*11-14:COOMe, and *E*10*E*11-14:COOMe (57:11:11:1:7:14)^21^ (**B**) *Z*9*E*12-14:COOMe and *Z*9-14:COOMe (1:1), (**C**) *Z*9*E*11-14:COOMe and *E*10*E*12-14:COOMe (1:1), and (**D**) *Z*9*E*12-14:COOMe and *Z*9*E*11-14:COOMe (1:1). *Spodoptera* pgFAR essentially reduces the 14:acid, 16:acid and *Z*9-16:acid compounds that are naturally present in the yeast to alcohol.

*S. littoralis* uses the pheromone blend of (*Z,E*)-9,11-tetradecadienyl acetate (*Z*9*E*11-14:OAc), *Z*9-14:OAc, *E*11-14:OAc, 14:OAc, *Z*11-14:OAc, and (*E,E*)-10,12-tetradecadienyl acetate (*E*10*E*12-14:OAc) at a ratio of 57:11:11:1:7:14^21^. SlitpgFAR II from *S. littoralis* was recently characterized, but the reductase specificity for shaping the pheromone blend has not been demonstrated^18^. Hence, we attempted to perform a functional assay in which SlitpgFAR II-transformed yeast cells were supplemented with the above-mentioned precursor FAME compounds at the indicated ratios. We obtained similar quantities of *Z*9*E*11-14:OH (28%), *E*11-14:OH (25%), *Z*11-14:OH (27%) and *Z*9-14:OH (20%) and a lower concentration of *E*10*E*12-14OH (Fig. 6A). The results indicate the different enzyme activities of the *S. exigua* and *S. littoralis* reductase systems toward di-unsaturated pheromone precursors. The former is selective for the *Z*9-14:acid, whereas the latter is selective for the *Z*9*E*11-14:acid.

Because we obtained selectivity in the *S. littoralis* pgFAR system for C_14_ mono acyls and *Z*9*E*11-14:acids, which contradicts the results reported by Carot-Sans *et al*.^18^, we performed additional substrate specificity assays with *S. littoralis* pgFAR II. In a functional assay with SlitpgFAR II-transformed yeast cells supplemented with methyl esters of the *Z*9-14:acid and *Z*9*E*11-14:acid at a ratio of 1:1, we obtained a significantly higher concentration of *Z*9-14:OH (Fig. 6B). We performed another functional assay with a ratio of *Z*9*E*11-14:COOMe to *E*10*E*12-14:COOMe equal to 1:1 in yeast selective media, and both compounds were reduced to the corresponding alcohols. The concentration of *Z*9*E*11-14:OH (*S. littoralis* major component) was similar to that of *E*10*E*12-14:OH (Fig. 6C). To confirm the similarity of the *S. exigua* and *S. littoralis* pgFAR activities, we performed a functional assay with SlitpgFAR II-transformed yeast cells supplemented with an equal concentration of *Z*9*E*12-14:COOMe (*S. exigua* major precursor) and *Z*9*E*11-14:COOMe (*S. littoralis* major precursor). *S. littoralis* pgFARII preferentially converted the *Z*9*E*12-14:acid to the corresponding alcohol (Fig. 6D).

### SexpgFAR II is selective and semi-selective for C_14_ mono- and di-unsaturated precursors, respectively, and SexpgFAR I is highly selective for C_16_ acyls

**Figure 7:**
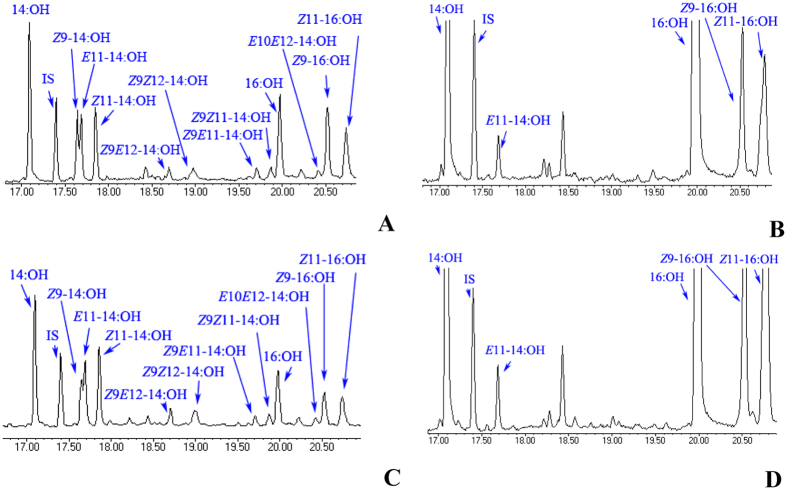
(**A–D**) Total ion chromatogram (TIC) showing the fatty alcohol products extracted from the yeast cells. The yeast (*InvSc1*) were transformed with the pgFAR construct and supplemented with a blend of equal concentrations (0.5 mM total) of *Z*11-16:COOMe, C14:COOMe, *E*11-14:COOMe, *Z9*-14:COOMe, *Z*11-14:COOMe, *Z*9*Z*11-14:COOMe, *Z*9*E*12-14:COOMe, *Z*9*E*11-14:COOMe, *E*10*E*12-14:COOMe and *Z*9*Z*12-14:COOMe in 5 mL of selective media. *Spodoptera* pgFAR reduces the 14:acid, 16:acid and *Z*9-16:acid compounds that are naturally present in the yeast. (**A**) SexpgFAR II, (**B**) SexpgFAR I, (**C**) SlitpgFAR II and (**D**) SlitpgFAR I.

Among the 18 *Spodoptera* spp. pheromones identified, most species use *Z*9*E*12-14:OAc as a major pheromone compound, followed by *Z*9*E*11-14:OAc and *E*10*E*12-14:OAc (Table S1). Only four *Spodoptera* spp. (*S. frugiperda, S. pectinicornis*, *S. praefica* and *S. triturate*) exclusively use the C12 and C14 mono-unsaturated fatty acetate compounds as a pheromone (specifically, *Z*7-12:OAc and *Z*9-14:OAc). We performed a functional assay with SexpgFAR II-transformed yeast cells supplemented with equal concentrations of five mono-unsaturated and di-unsaturated compounds (Fig. 7) in 5 mL of selective media. *Z*9-14:OH, 14:OH, *Z*11-14:OH and *E*11-14:OH production was significantly increased compared with di-unsaturated alcohol production (Fig. 7A). We also performed a functional assay with SlitpgFAR II by supplementing yeast cells with equal concentrations of 10 precursor compounds and obtained the results similar to those obtained with SexpgFAR II (Fig. 7C). In another set of functional assays, SexpgFAR I-transformed yeast cells were supplemented with equal concentrations of all 10 compounds, and as expected, we obtained only 14:OH, *Z*11-16:OH and a trace amount of *E*11-14:OH (Fig. 7B). The same functional assay experiments were repeated with SlitpgFAR I-transformed yeast cells and yielded the same results (Fig. 7D). These findings indicate that the substrate specificities of pgFAR I in *S. exigua* and *S. littoralis* are similar.

The yeast functional assay (multi-substrate) results for SexpgFAR II also showed that all of the monounsaturated methyl esters were converted to alcohols at approximately the same level; however *Z*9-14:OH presented the highest production, followed by *Z*11-14:OH and *E*11-14:OH (Fig. 7A). We obtained lower quantities of the fatty alcohols derived from the di-unsaturated compounds (Fig. 7A). This study indicates that SexpgFAR II is selective for C14 and C16 mono-unsaturated fatty acyls. Thus, SexpgFAR II is semi-selective for reduction of the *Z*9*E*12-14:acid and highly selective for reduction of mono-unsaturated C14-fatty acids to alcohols. Nevertheless, *Spodoptera* pgFAR I is unable to reduce the C14 mono- and di-unsaturated pheromone precursors, with the exception of *E*11-14:COOMe, and is selective for C16 fatty acid compounds.

### A yeast functional assay revealed that both SexpgFAR I and SexpgFAR II cannot reduce C_10_ and C_12_ fatty acid precursors

Among the 18 *Spodoptera* spp. reported, *S. frugiperda, S. pectinicornis* and *S. praefica* use saturated and mono-unsaturated C12 acetate esters as pheromones^9^. Through a single-substrate assay, we tested the substrate chain length preference of SexpgFAR I and SexpgFAR II by supplementing the yeast cells with 0.5 mM (*Z*)-5-decenyl acid Me (*Z*5-10:COOMe) and (*Z*)-7-dodecenyl acid Me (*Z*7-12:COOMe). None of these compounds were converted into the corresponding alcohols (results not shown). The results indicate that SexpgFAR I and SexpgFAR II are unable to reduce any fatty acyl substrates shorter than C_14_.

## Discussion

**Figure 8:**
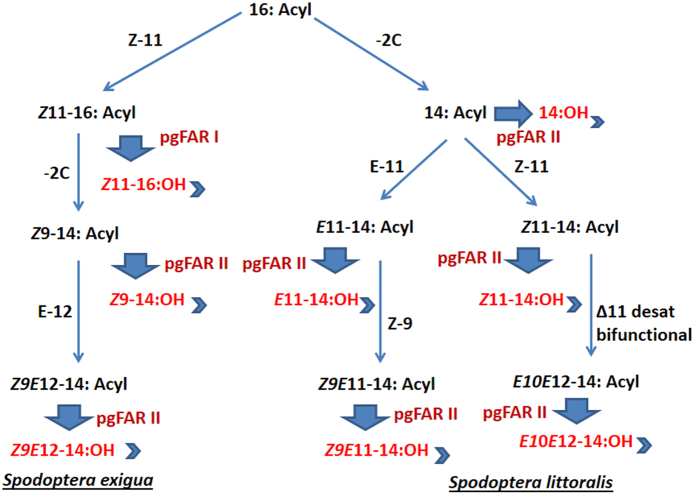
*Spodoptera exigua* and *S. littoralis* pheromone biosynthesis pathway for the production of pheromone precursors. The *de novo* biosynthesis of all precursors starts from palmitoyl-CoA (16:acyl). In *S. exigua*, a Δ11 desaturase catalyzes the production of (*Z*)-11-hexadecenyl [(*Z*)-11–16:acyl], which undergoes one cycle of β-oxidation to produce (*Z*)-9-tetradecenyl [(*Z*)-9–14:acyl]^22^. A Δ12 desaturase catalyses (*Z*)-9–14:acyl to produce (*Z,E*)-9,12- tetradecadienyl acyl (*Z*9*E*12-14:acyl). In *S. littoralis*, 16:acyl undergoes one cycle of β-oxidation to produce myristoyl-CoA (14:acyl). A Δ11 desaturase acts on 14:Acyl to produce (*E*)- and (*Z*)-11-tetradecenoyl [(*E*) + (*Z*)-11–14:acyl]^21^. A Δ9 desaturase acts on (*E*)-11–14:acyl to produce (*Z,E*)-9,11- tetradecadienyl acyl (*Z*9*E*11-14:acyl), and a bifunctional Δ11 desaturase acts on (*Z*)-11–14:acyl to produce (*E,E*)-10,12- tetradecadienyl acyl (*E*10*E*12-14:acyl)^50^. Each compound undergoes a reduction step catalysed by pgFAR I and pgFAR II to produce the corresponding fatty alcohol derivatives. Finally, an acetyltransferase catalyses the conversion of the fatty alcohol precursors into the corresponding acetate esters (shown with an arrowhead).

We identified two active FARs that are exclusively expressed in the PG and exhibit specificity for C_16_ and C_14_ acyls, respectively, and demonstrate that both FARs are involved in the *Spodoptera* pheromone biosynthesis pathway to produce different precursor alcohol compounds. Our yeast functional assay data confirm that pgFAR I can primarily reduce C_16_ fatty acyl precursors, whereas pgFAR II was found to be involved in the reduction of a broad range of saturated and unsaturated C_14_ acyls. FAR could reduce neither C_10_ nor C_12_ fatty acyl precursors. The discovery of a C_16_-specific function of FAR I may not be consistent with the function of the previously identified FARs from corn borer moths^7^, small ermine moths^19^ and heliothine moths^17^ that produce a broad range of alcohols. However, the degree of substrate specialization and the close relationship between SexpgFAR I and *B. anynana* FAR^26^ led us to assume that the pheromone biosynthesis pathway is shared between moths and butterflies. Furthermore, SexpgFAR II, which is similar to all of the broad-range FARs mentioned above, is capable of reducing C_14_, adding to the degree of specialization, as observed with the production of *Z*9*E*12-14:OH in *S. exigua* and *Z*9*E*11-14:OH in *S. littoralis.* The enzymatic activity and substrate specificity identified in this study clearly suggest that the *Spodoptera* female sex pheromone components are produced *via de novo* common biosynthetic routes^13^ that involve both FARs (Fig. 8). We identified eight duplicate FAR genes from the pheromone gland of *S. exigua*, found that SexpgFAR I and SexpgFAR II belong to the Lepidoptera-specific pgFAR lineage and demonstrated that these two genes encode specialized pheromone biosynthetic reductases. A recent study of the evolution of FAR genes in plants and animals clearly indicated that insects have undergone large expansions relative to the other taxa examined, which is consistent with a hypothesis that FAR is a multigene family that evolved through the birth-death evolution^35^. Extensive studies on the evolutionary pattern of FAR genes in plants and animals show considerable losses and gains of novel functions as a result of gene duplication events, and insects gained a substantial number of FAR genes when they diverged from other animals^35^. There are two reductase genes present in the vertebrate genomes, whereas more than a dozen are present in Lepidoptera^14^,^36^. Among these genes, a sub-group of FARs gained the ability to convert palmitic acid (C16: acid) into hexadecanol (C16:OH). These FARs are involved in pheromone biosynthesis in moths^16^ and butterflies^26^. In moths, FAR expansions have facilitated the adaptive evolution of a variety of specialized functions involving the precursors that serve as substrates for reductase genes. In *S. exigua* and *S. littoralis*, the presence of mono- and di-unsaturated C_14_ and C_16_ fatty acyl precursors might prime the expansion of two pgFARs in these species.

Several pgFAR orthologs that are specific to C16 acyl precursors are reported to produce traces of C14 saturated and unsaturated fatty alcohols, as reported in *B. mori*^16^ and *B. anynana*^26^. Hence, we hypothesize that the production of trace amounts of C14:OH and *E*11-14:OH by SexpgFAR I may result from an ancestral feature that is capable of reducing C_14_ and C_16_ acyl precursors. However, the ancestral activity was less specialized and reduced C14:acyl and *E*11-14:acyl. To confirm this hypothesis, we performed multi-substrate functional assays with BmopgFAR-transformed yeast cells supplemented with 10 different precursors as a positive control (Fig. S5). Similarly to SexpgFAR I, BmorpgFAR was unable to reduce the di-unsaturated C_14_ fatty acid precursors. However, trace amounts of *E*11-14:OH, *Z*11-14:OH and a large amount of *Z*11-16:OH were observed in the yeast cell extract (Fig. S5). Another functional assay with a rare C_16_ precursor, *E*14-16:acid, revealed that SexpgFAR I and BmopgFAR but not SexpgFAR II can reduce the *E*14-16:acid. These results indicate the functional similarity of *B. mori*, *S. exigua* and *S. littoralis* FAR I, which are specialized to reduce C16 acid derivatives. The single-substrate functional assay confirmed that SexpgFAR II is involved in the reduction of a broad range of saturated and unsaturated C_14_ acyls and that SexpgFAR I could reduce only *Z*11-16:acyls. Many *Spodoptera* species use *Z*11-16:OAc as a minor pheromone compound (Table S1). Several orthologous pgFARs that are specific for C_14_ acyl precursors are reported to produce traces of C_16_ saturated and unsaturated fatty alcohols^11^,^19^. Therefore, the production of trace amounts of *Z*11-16:OH derivatives by SexpgFAR II may reflect an ancestral feature. These preadaptations of the reductase system have also been reported in *O. furnacalis*, which uniquely possesses a ∆12-tetradecenyl precursor as a consequence of the activity of ∆14 desaturases^33^,^37^; however, pgFAR can reduce *E/Z*11-16 acyls^11^.

The yeast functional assay results show that SexpgFAR II presents activity toward a wide range of fatty acyl precursors. Although we did not achieve the same blend ratio as was previously reported^21^, a preference of SlitpgFAR II toward the *Z*9*E*11-14:acid was observed, and the results contradict those published previously^18^. One reason for this discrepancy may be that the pheromone blend ratio reported by Munoz *et al*.^21^ involved a PG extract lipid analysis, and an SPME analysis of *S. littoralis* has not been performed. In contrast, an SPME analysis of *S. exigua* PG has revealed a ratio that differs from that obtained through a gland extract analysis^22^. Several insect pheromone blend ratios were recently revised through an SPME analysis (for example, *Batrachedra amydraula*)^38^,^39^. Hence, we reiterate that the selectivity of SlitpgFAR II favors the *Z*9*E*11-14:acid. In contrast to the reported PG extract ratio of *E*10*E*12-14:OAc (14%), we found significantly lower quantities of *E*10*E*12-14:OH (>4%) (Fig. 6A). The explanation for the SPME analysis also applies in this case. However, we cannot rule out the selectivity of desaturases and acetyltransferases in shaping the final pheromone blend in *Spodoptera.* Similarly to yponomeutid and heliothine pgFARs^17^,^19^, *Spodoptera* pgFAR II accepts a broad range of substrates, suggesting that their function in the PG evolved in the genus prior to species diversification. Hence, evolutionary divergence probably occurred upstream of the desaturases or downstream of the acetyltransferases. In this case, the production of a specific blend of pheromone compounds depends on the available fatty acyl precursors or the selectivity of the acetyltransferase. The acetyltransferase has not been characterized at the molecular level in any insect^13^,^40^,^41^,^42^. Additional comparative functional analyses of the desaturase, reductase and acetyltransferase activities must be conducted to prove this hypothesis.

The multi-substrate functional assay of SexpgFAR II with different blend ratios of *Z*9-14:COOMe and *Z*9*E*12-14:COOMe resulted in higher *Z*9-14:OH production, indicating the selectivity of the *S. exigua* pgFAR system. We obtained pheromone blend ratios of the major pheromone compounds that were similar to those previously reported^22^. Our multi-substrate functional assays proved that both *Spodoptera* pgFARs are selective for the *Z*9-14:acid, and *S. exigua* and *S. littoralis* exhibit a preference for its major precursors: *Z*9-14:acid, *Z*9*E*12-14:acid and *Z*9*E*11-14:acid, respectively. Of the 18 identified *Spodoptera* spp. pheromones, *Z*9-14:OAc is a major pheromone component, and 14 *Spodoptera* spp. use *Z*9*E*12-14:OAc in their pheromone blends (Table S1). Our functional assays highlight the fact that all of the present-day *Spodoptera* pgFARs have the ability to reduce substantial quantities of these two compounds, as demonstrated for *S. exigua* and *S. littoralis* in our study (Figs 5 and 6). Although *S. littoralis* does not use *Z*9*E*12-14:OAc as a pheromone compound, several studies have demonstrated the presence of this compound in the PG extract^43^,^44^. The results indicate that both *S. exigua* and *S. littoralis* are selective for *Z*9*E*12-14:acid among all di-unsaturated C_14_ fatty acids. We hypothesized the existence of a functional site or domains in the FARs that define this property, which is highly conserved among all *Spodoptera* pgFARs. Previous studies of heliothine pgFARs demonstrated a preference for *Z*9-14:acid compounds^17^. The functional ability of *S. exigua* pgFAR II to reduce *Z*9-14:acid might be an ancestral feature that is retained in noctuid moths. However, the ability of the *Spodoptera* pgFAR to reduce the *Z*9*E*12-14:acid (or *Z*9*E*11-14:acid) is a characteristic of this genus. Until now, no other Lepidoptera pgFARs have been reported to exhibit multifunctional activity toward mono- and di-unsaturated pheromone precursors^7^,^11^,^14^,^15^,^16^,^17^,^19^.

A single pgFAR with broad selectivity for C16 fatty acyl precursors with a distinct substrate specificity for *E*10*Z*12-16:acid has been reported in *B. mori*^16^. Highly selective pgFARs that are unable to reduce any precursors other than the natural pheromone precursors, *E*11-14:acid and *Z*11-14:acid, have been reported in *O. nubilalis*^7^. In contrast, the orthologous reductases from *Yponomeuta* spp., *Agrotis* spp. *Heliothis* and *Helicoverpa* spp. reduce a broad range of C_12_, C_14_ and C_16_ acyl precursors but present clear specificity for the natural C_14_ pheromone precursors^15^,^17^,^19^. In the present study, we identified two pgFARs in *S. exigua* and *S. littoralis*. pgFAR I in both species possesses similar functional activity, and its function might have evolved before radiation within the *Spodoptera* genus. We reiterate that the *Spodoptera* pgFAR I selectively recognizes the chain length of the substrates for pheromone biosynthesis, whereas pgFAR II accepts a broad range of substrates. pgFAR II is selective for the major pheromone precursors (*Z*9-14:acyl and *Z*9*E*12-14:acyl for *S. exigua*; *Z*9*E*11-14:acyl for *S. littoralis*), which unequivocally supports the hypothesis that the *S. exigua* and *S. littoralis* pgFARs play important roles in shaping the final pheromone blend ratio. Overall, our findings demonstrate that two biosynthetic pgFARs are involved in *Spodoptera*. pgFAR II exhibits broad selectivity and acts on C_14_ mono- and di-unsaturated precursors, and pgFAR I exhibits narrow selectivity and acts on *Z*11-16:acyl to produce the moth pheromone signals. The mechanism through which *Spodoptera* pgFARs coordinate the reduction of the mono- and di-unsaturated C_14_ and C_16_ precursors remains to be elucidated. In most *Spodoptera* species, the precursor and pheromone component ratios exhibit significant disparities (Table S1), and the pgFAR substrate preference is necessary to explain the production of species-specific pheromone blends. Our yeast functional assay revealed that both FARs cannot reduce C_10_ and C_12_ acyl precursors, which indicates that another potentially active FAR candidate may be involved in the reduction pathways in *S. frugiperda. S. praefica* and *S. pectinicornis* (see Table S1). Our study of the two pgFARs in *Spodoptera* will form a basis for the identification of FARs in other *Spodoptera* moths. Our major findings are that a subgroup of FARs (pgFAR I) dedicated to the reduction of C16:acid is functionally conserved in moth and butterfly lepidopteran lineages and that a multifunctional pgFAR II acts on the mono and di-unsaturated C_14_:acid precursors that are collectively involved in *Spodoptera* pheromone biosynthesis. Our study opens the door for a new area of research aiming to disclose the evolutionary changes associated with C_14_-specific and C_16_-specific FARs in moth pheromone biosynthesis.

## Materials and Methods

### Chemicals

The chemicals and authentic standard compounds mentioned in the supporting information (SI) were purchased from Pest Control of India Private Limited (Mumbai, India) and Pherobank (Netherlands). The compounds used for the functional assays were diluted in 95% ethanol (0.5 mM), and the standard compounds were diluted in *n*-hexane (250 ng/μL).

### Insects

*Spodoptera exigua* and *S. littoralis* pupae were originally collected from a vegetable garden [Al-Kharj, (24.1500°N, 47.3000°E) Saudi Arabia], and laboratory cultures were maintained on an artificial diet^45^. Adult moths were collected from private properties for which permits were not required. *S. exigua* and *S. littoralis* are not endangered or protected species. The emerged females were separated daily prior to reaching scotophase, and their pheromone glands were dissected^14^.

### PCR amplification and cloning of the pgFAR transcripts

We extracted the total RNA from the pooled pheromone glands of 0- to 3-day-old virgin *S. exigua* females using an RNeasy isolation kit (Qiagen). First-strand cDNAs were prepared using ArrayScript reverse transcriptase (Life Technologies). We created a dataset of FARs using EST, as previously reported^14^, and the degenerate primers are listed in Table S2. Briefly, 500 ng of the PG cDNAs was used to amplify the FARs, and the PCR product was gel purified (Promega), cloned into a pGEMt vector and transformed into chemically competent *Escherichia coli* JM109 cells (Promega). A total of 400 clones (100 for each degenerate PCR, see Table S2) was selected. The plasmid DNA was purified, and sequencing reactions were performed using a BigDye terminator kit v3.1 (Applied Biosystems) and subsequently analyzed on an ABI PRISM 3500 genetic analyzer (Life Technologies). The sequences were identified through BLASTx searches using the *S. exigua* FAR sequences as the queries. The 5′ and 3′ cDNA specimens were prepared with the SMART RACE amplification kit (Clontech) according to the manufacturer’s instructions. Gene-specific primers were designed (Table S2) and used in the PCR reactions to amplify the PCR products, which were gel-purified and ligated into the pGEM-T easy vector (Promega). The ligation products were transformed into *Escherichia coli* JM109 chemically competent cells (Promega). Plasmid DNA specimens were purified, sequenced, and analyzed using an ABI 3500 genetic analyzer. Separate sequencing reactions were established for primer walking to obtain the full-length pgFAR sequences, and the data were compiled using the BioEdit program^46^.

### Tissue-specific expression analysis

The total RNA from the entire thorax and abdomen (without PG) of 3-day-old female *S. exigua* moths (n = 3) and from the entire thorax and abdomen of male moths (n = 3) was prepared using an RNeasy mini kit (Qiagen). The first-strand cDNAs were synthesized from 1 μg of the total RNA using an ArrayScript RT kit (Life Technologies). Touchdown PCR was performed using 200 ng of the cDNA template and primer sets designed for the specific amplification of each FAR-like sequence (Table 2). The conditions were 94 °C for 1 min, 10 cycles of 94 °C for 30 s, 60 °C for 1 min (reduced by 1 °C/cycle), and 72 °C for 1 min, 20 cycles of 94 °C for 30 s, 50 °C for 1 min and 72 °C for 1 min, and a final extension at 72 °C for 7 min. The PCR products were electrophoretically separated on a 2% agarose gel.

### Analysis of the pgFAR transcript abundance through a quantitative PCR analysis

Three PGs were dissected from 2- to 3-day-old female *S. exigua* moths at mid-scotophase. Total RNA was extracted (Qiagen), and cDNAs were synthesized using MultiScribe™ Reverse Transcriptase (Life Technologies). Three biological cDNA samples were prepared, and quadruplicate 25-μL quantitative PCR reactions for each transcript (SexpgFAR I, SexpgFAR II and β-actin) were performed on an ABI 7500 instrument (Life Technologies). The template was 100 ng of PG cDNA, and SexpgFAR I, SexpgFAR II, and β-actin primers at a concentration of 10 μM were used (Table S2) (Tm = 60 °C). Premier Primer software (Biosoft International) and the Power SYBR Green qPCR MasterMix were used (Life Technologies). The amplification efficiency (E) of each primer pair was initially assessed from serial dilutions of the cDNAs, and the raw cycle threshold values (Ct) were averaged from the quadruplicate reactions. The β-actin RNA was used for normalization, and all relative expression levels were calculated using the ABI 7500 program (Life Technologies). The fluorescence background baselines and amplification thresholds were calculated automatically. The fold changes in expression levels were calculated against a normalized (β-actin) sample and subjected to one-way analysis of variance (ANOVA). Means were compared using Tukey’s HSD (honestly significant difference) test in the SPSS program^47^.

### Phylogenetic analysis

The *S. exigua* and *S. littoralis* FAR nucleotide sequences were used as queries for BLASTx searches in the GenBank database, and the nucleotide and amino acid sequences from different insect species were retrieved to construct a phylogenetic tree. Similarity analyses of the DNA and protein sequences were conducted, and multiple-sequence alignment was performed using the MUSCLE program^48^. A maximum likelihood analysis was performed, and a dendrogram was constructed using MEGA v6.0^49^.

### Functional gene expression and assay

A yeast expression system (Invitrogen) was used to functionally express the *S. exigua* and *S. littoralis* pgFAR transcripts according to a previously described protocol^7^,^19^. We used pYES2.1/V5-His TOPO (Invitrogen) as a shuttle vector for the pgFAR transcripts, and the plasmids were transformed into *Saccharomyces cerevisiae* (INVSc1 strain, Invitrogen) according to the protocol listed in the SI Materials and Methods. For each test sample in the functional assay, we included a negative control (vector only) and a positive control (*B. mori* pgFAR)^16^. All oligonucleotide primers and a synthetic gene representing the ORF of *B. mori* pgFAR were purchased from Integrated DNA Technologies (Belgium).

### pgFAR substrate preference analysis

The pgFAR yeast transformants were supplemented with different ratios of the precursors that were added at a total concentration of 0.5 mM. Single- and multi-substrate assays with different blend ratios (mentioned in the results) were performed using three replicates for each experiment as well as a negative control (vector only) and a positive control (*B. mori* pgFAR)^16^. The incubation and extraction protocols and the gas chromatography coupled to mass spectrometry (GC-MS) analysis were performed as described in the SI Materials and Methods^7^,^19^. The final fatty alcohol production was calculated as previously described^7^,^11^. The standard deviations (SDs) and standard errors of the mean (SEMs) were calculated using MS Excel. The significant differences in alcohol production in each functional assay were calculated *via* ANOVA tests followed by Tukey’s test, and homogeneity-of-variance tests were performed using the SPSS program.

## Additional Information

**Accession codes:** The sequences reported in this paper have been deposited in the GenBank database (Accession Nos KF805977-KF805983 and KR781119-KR781121).

**How to cite this article**: Antony, B. *et al*. Two *fatty acyl reductases* involved in moth pheromone biosynthesis. *Sci. Rep.* **6**, 29927; doi: 10.1038/srep29927 (2016).

## Supplementary Material

Supplementary Information

## Figures and Tables

**Table 1:** Percentages of *fatty acyl reductase* (FAR) nucleotide and amino acid identity and similarity.

**FAR (GenBank Acc. Nos.)**	**SexpgFAR I**		**SlitpgFAR I**		**SexpgFAR II**		**SlitpgFAR II**	
	**nt**	**aa**	**nt**	**aa**	**nt**	**aa**	**nt**	**aa**
BmorpgFAR (NM_001043502)	51.30 (60.27)	38.99 (61.87)	50.46 (59.72)	40.21 (62.37)	53.04 (63.11)	41.62 (66.38)	52.23 (62.5)	41.18 (66.05)
OscpgFAR E (EU817405)	46.88 (55.93)	32.46 (54.68)	46.0 (55.48)	35.06 (55.01)	44.68 (55.32)	31.71 (54.51)	44.39 (55.09)	31.71 (54.34)
OscpgFAR Z (AB506111)	46.73 (55.82)	30.93 (53.84)	46.0 (55.48)	33.98 (54.18)	44.54 (55.20)	30.17 (54.18)	44.39 (55.09)	30.39 (54.01)
YevpgFAR (ADD62439)	51.70 (60.38)	44.98 (64.04)	50.88 (59.77)	45.65 (64.71)	50.81 (61.78)	43.42 (66.55)	49.85 (61.05)	42.76 (65.71)
HaspgFAR (AFD04727)	54.26 (62.45)	44.73 (64.88)	55.28 (63.23)	45.61 (64.54)	71.35 (77.66)	74.22 (86.45)	70.91 (77.32)	73.34 (85.28)
HarpgFAR (AFD04728)	54.45 (62.72)	46.37 (65.05)	55.4 (63.45)	47.03 (64.71)	70.91 (77.54)	72.68 (86.12)	71.06 (77.66)	72.46 (84.94)
AsepgFAR (AGP26039)	53.74 (61.94)	44.10 (64.21)	53.74 (61.94)	44.54 (63.54)	71.5 (77.66)	68.72 (84.44)	71.86 (77.93)	69.16 (84.28)
Ban-wFAR 1 (AGD98718.1)	55.63 (62.84)	50.33 (66.55)	54.57 (62.06)	48.98 (65.21)	56.98 (66.12)	49.66 (72.9)	55.48 (65.01)	49.43 (72.07)
Ban-wFAR 2 (AGD98719)	53.40 (60.27)	45.96 (62.87)	53.05 (60.27)	45.45 (61.87)	55.82 (61.94)	48.89 (67.05)	55.09 (61.39)	48.23 (66.55)
HsaFAR (AY600449)	45.72 (44.84)	33.11 (46.48)	45.21 (44.67)	34.41 (46.32)	46.15 (46.62)	33.92 (50.33)	46.0 (46.51)	34.14 (49.66)
SchiFAR (AF149917)	41.95 (45.68)	27.45 (43.31)	41.75 (45.73)	27.92 (43.47)	42.85 (48.18)	24.88 (45.15)	41.75 (47.35)	24.22 (45.15)

The pairwise sequence identity and similarity from the Clustal W multiple sequence alignments^49^ were calculated using the SIAS program (http://imed.med.ucm.es/Tools/sias.html).

Numbers in parenthesis are the similarity values. nt: nucleotide; aa: amino acid. SexpgFAR: *Spodoptera exigua* pgFAR; SlitpgFAR: *S. littoralis* pgFAR; BmorpgFAR: *Bombyx mori* pgFAR; OscpgFAR E: *Ostrinia scapulalis* (*E*-strain) pgFAR; OscpgFAR Z: *Ostrinia scapulalis* (*Z*-strain ) pgFAR; YevpgFAR: *Yponomeuta evonymellus* pgFAR; HaspgFAR: *Helicoverpa assulta* pgFAR; HarpgFAR: *H. armigera* pgFAR; AsepgFAR: *Agrotis segetum* pgFAR; Ban-wFAR 1: *Bicyclus anynana* FAR 1; Ban-wFAR 2: *B. anynana* FAR 2; HsaFAR: *Homo sapiens* FAR; SchiFAR: *Simmondsia chinensis* FAR.

**Table 2:** Summary of the pgFAR activities toward different substrates.

**Substrate tested**	**SexpgFAR I**	**SlitpgFAR I**	**SexpgFAR II**	**SlitpgFAR II**
*Saturated fatty acids*				
C14:Acyl/C16:Acyl	C16:Acyl	C16:Acyl	C14:Acyl	C14:Acyl
	C14:Acyl	C14:Acyl	C16:Acyl	C16:Acyl
*Monounsaturated fatty acids*				
*Z*11-16:Acyl	*Z*11-16:Acyl	*Z*11-16:Acyl	*Z*12-14:Acyl	Z9-14: Acyl
*E*11-14:Acyl	*E*14-16:Acyl	*E*14-16:Acyl	*Z*9-14: Acyl	*Z*12-14:Acyl
*Z*9-14:Acyl	*E*11-14: Acyl	*E*11-14: Acyl	*E*11-14: Acyl	*E*11-14:Acyl
*E*12-14:Acyl	*Z*11-14: Acyl	*Z*11-14: Acyl	*E*12-14:Acyl	*Z*11-14:Acyl
*Z*12-14:Acyl			*Z*11-16:Acyl	*E*12-14:Acyl
*Z*7-12:Acyl*			*Z*11-14: Acyl	*Z*11-16:Acyl
*E*14-16:Acyl				
*Z*5-10:Acyl*				
*Z*11-14:Acyl				
*Diunsaturated fatty acids*				
*Z*9*Z*11-14:Acyl	—	—	*Z*9*E*12-14:Acyl	*Z*9*Z*12-14:Acyl
*Z*9*E*12-14:Acyl			*Z*9*Z*12-14:Acyl	*Z*9*E*12-14:Acyl
*Z*9*E*11-14:Acyl			*Z*9*E*11-14:Acyl	*Z*9*E*11-14:Acyl
*E*10*E*12-14:Acyl			*E*10*E*12-14:Acyl	*E*10*E*12-14:Acyl
*Z*9*Z*12-14:Acyl			*Z*9*Z*11-14:Acyl	*Z*9*Z*11-14:Acyl

The total fatty alcohol production from three biological replicates was quantified^11^, and based on the quantity of the fatty alcohols produced in yeast assays, the preferred substrates were arranged from highest to lowest in each row.

Not reducing to alcohol; —indicate no enzyme activity.
