# Supplementary material for: Two fatty acyl reductases involved in moth pheromone biosynthesis
Source: Sci Rep. 2016 Jul 18;6:29927. doi: 10.1038/srep29927 (PMC4947906; doi:10.1038/srep29927)
Supplement: Supplementary Information [file srep29927-s1.pdf]

Supporting information

Two *fatty acyl reductases* involved in moth pheromone biosynthesis

Antony, B., Ding, B-J., Moto, K-I., Aldosari, S.A. and Aldawood, A.S.

Fig. S1. Quantitative PCR results showing relative expression of SexpgFAR I and SexpgFAR II in the pheromone gland of 2-3d old female moth at mid-scotophase. The actin gene expression is used to normalize the gene expression level in the PG and used as control gene. In the analysis of the relative pgFAR expression change in, the actin gene was taken as the calibrator. The mean relative expression scores were calculated from the raw cycle threshold (ΔC_T) values. The transcripts with same letters are not significantly different ($P < 0.05$).

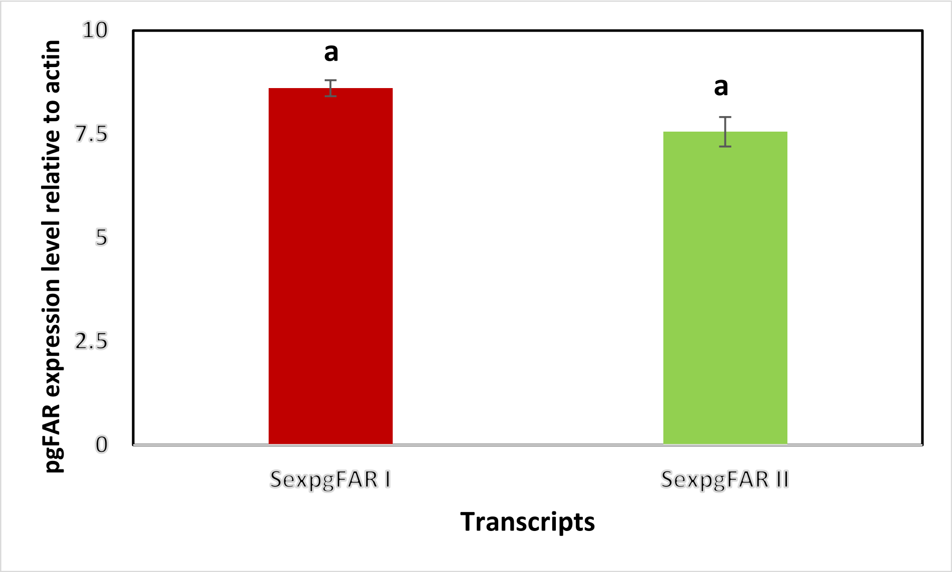

Fig. S2. Functional assay and GC-MS analysis of yeast (*InvSc1*) transformed with the pgFAR construct and supplemented with 0.5 mM C14:COOMe and C16:COOMe (**A**), *Z*11-16:COOMe (**B**) and *E*14-16:COOMe (**C**). The total ion chromatogram (TIC) shows the fatty alcohol products extracted from the yeast cells after a 48 h incubation at 30 °C and 300 rpm. Yeast cells transformed with an empty vector (negative control) or *B. mori* pgFAR (positive control)[16] ensured that the production of alcohol in yeast cells was due to the recombinant pgFAR gene. RT: retention time; IS: internal standard (250 ng 15:OAc). The authentic standards used in this assay were 16:OH, 14:OH, *Z*11-16:OH and *E*14-16:OH.

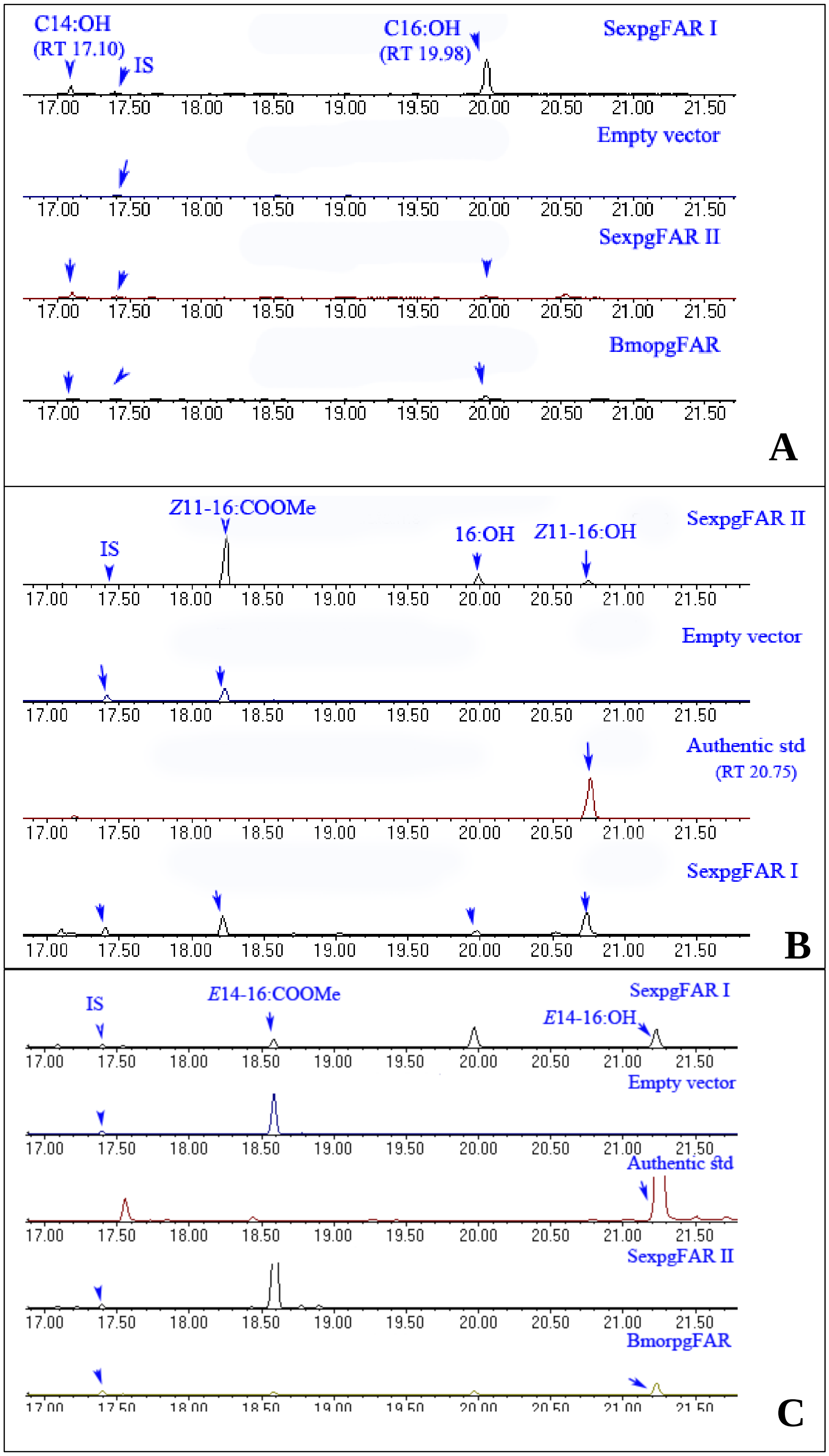

Fig. S3A-H. Functional assay and GC-MS analysis of yeast (*InvSc1*) transformed with the pgFAR construct and supplemented with 0.5 mM *E*11-14:COOMe (**A**), *Z*9-14:COOMe (**B**), *Z*11-14:COOMe (**C**), *Z*9*Z*11-14:COOMe (**D**), *Z*9*E*12-14:COOMe (**E**), *Z*9*E*11-14:COOMe (**F**), *E*10*E*12-14:COOMe (**G**) and *Z*9*Z*12-14:COOMe (**H**). Yeast cells transformed with the empty vector (negative control) ensured that the production of alcohol in yeast cells was due to the recombinant pgFAR gene. The authentic standards used in this experiment were *E*11-14:OH (**A**), *Z*9-14:OH (**B**), *Z*11-14:OH (**C**), *Z*9*Z*11-14:OH (**D**), *Z*9*E*12-14:OH (**E**), *Z*9*E*11-14:OH (**F**), *E*10*E*12-14:OH (**G**) and *Z*9*Z*12-14:OH (**H**).

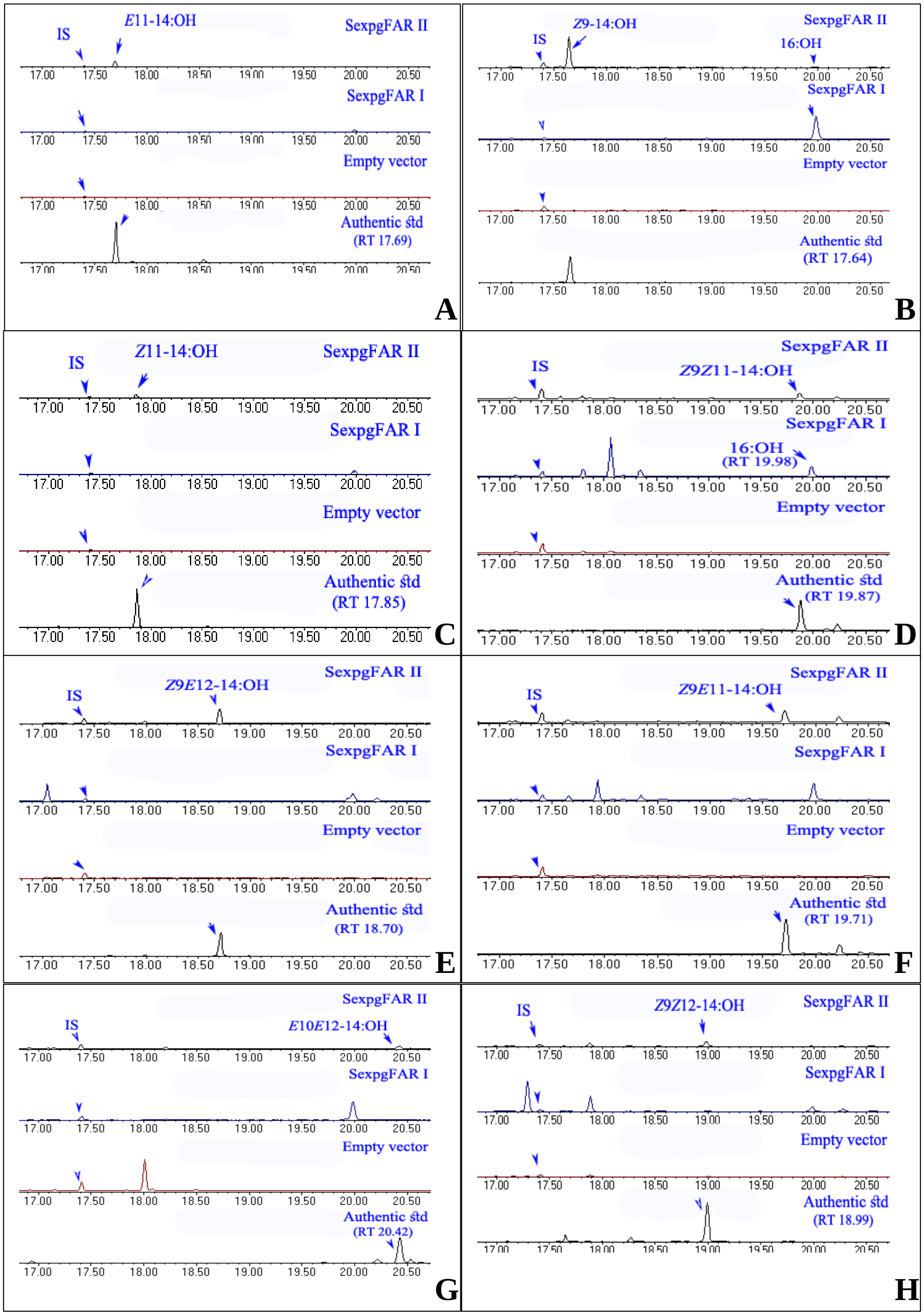
IS
E11-14:OH
SexpgFAR II
SexpgFAR I
Empty vector
Authentic std
(RT 17.69)
A
IS
Z9-14:OH
SexpgFAR II
16:OH
SexpgFAR I
Empty vector
Authentic std
(RT 17.64)
B
IS
Z11-14:OH
SexpgFAR II
SexpgFAR I
Empty vector
Authentic std
(RT 17.85)
C
SexpgFAR II
IS
Z9Z11-14:OH
SexpgFAR I
16:OH
(RT 19.98)
Empty vector
Authentic std
(RT 19.87)
D
SexpgFAR II
IS
Z9E12-14:OH
SexpgFAR I
Empty vector
Authentic std
(RT 18.70)
E
SexpgFAR II
IS
Z9E11-14:OH
SexpgFAR I
Empty vector
Authentic std
(RT 19.71)
F
SexpgFAR II
IS
E10E12-14:OH
SexpgFAR I
Empty vector
Authentic std
(RT 20.42)
G
IS
Z9Z12-14:OH
SexpgFAR II
SexpgFAR I
Empty vector
Authentic std
(RT 18.99)
H
17.00
17.50
18.00
18.50
19.00
19.50
20.00
20.50

Fig. S4. Functional assay and GC-MS analysis of yeast (*InvSc1*) transformed with the pgFAR construct and supplemented with 0.5 mM *E*12-14:COOMe and *Z*12-14:COOMe. The total ion chromatogram (TIC) shows the fatty alcohol products that were extracted from the yeast cells after a 48 h incubation at 30 °C and 300 rpm. Yeast cells transformed with the empty vector (negative control) ensured that the production of alcohol in the yeast cells was due to the recombinant pgFAR gene. RT: retention time; IS: internal standard (250 ng 15:OAc). The authentic standards used in this experiment were *E*12-14:OH and *Z*12-14:OH (Pherobank, The Netherlands).

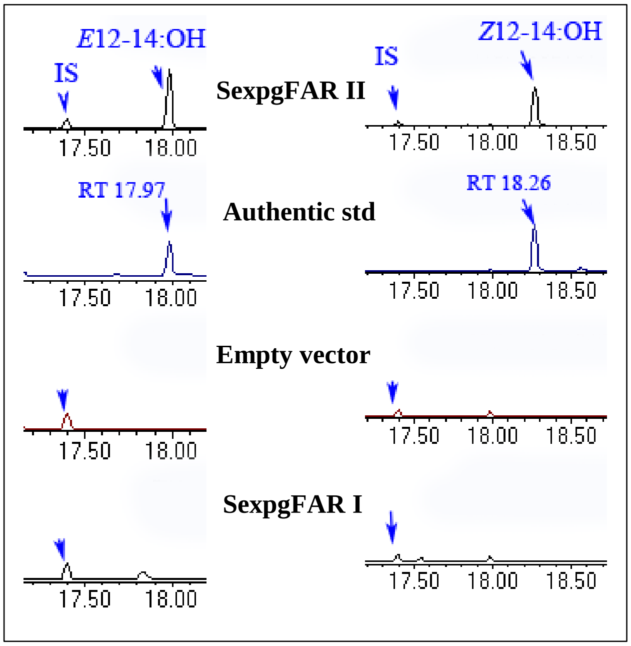

Fig. S5. Functional assay and GC-MS analysis of yeast (*InvSc1*) transformed BmopgFAR construct supplemented with a blend of equal concentration (0.5 mM total) of C14: COOMe, *E*11-14:COOMe, *Z*9-14:COOMe, *Z*11-14:COOMe, *Z*9*Z*11-14:COOMe, *Z*9*E*12-14:COOMe, *Z*9*E*11-14:COOMe, *E*10*E*12-14:COOMe and *Z*9*Z*12-14:COOMe in 5mL selective media. Total ion chromatogram (TIC) showing fatty alcohol products extracted from yeast cells after 48 h incubation at 30 °C, 300rpm. RT: retention time; IS: internal standard (250 ng 15:OAc). *B. mori* pgFAR reduces 14:acid and 16:acid compounds naturally present in the yeast, reduced to alcohol.

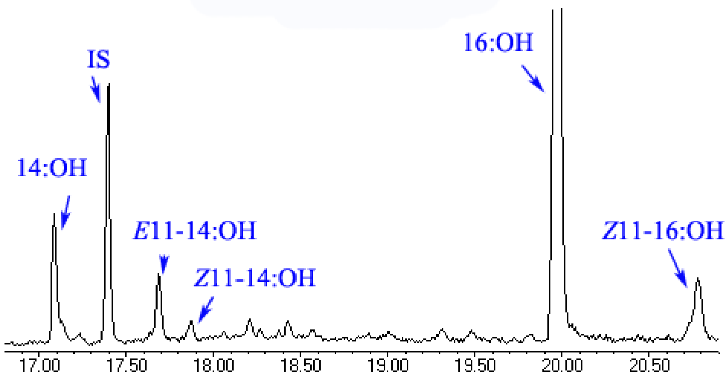

Table S1: List of the *Spodoptera* species with pheromone compounds identified.

No.	***Spodoptera* species**	**Pheromone compound**	**Reference**
1	*Spodoptera androgea*	*Z*9-14:OAc<br>*Z*9*E*12-14:OAc	[1]
2	*Spodoptera cilium* Guenée	*Z*9*E*12-14:OAc	[2]
3	*Spodoptera depravata* Butler	*Z*9*E*12-14:OAc<br>*Z*9-14:OAc	[3]
4	*Spodoptera descoinsi* Lalanne Cassou & Silvain	*Z*9-14:OAc<br>*Z*11-14:OAc<br>*Z*9*E*12-14:OAc<br>*E*9*E*12-14:OAc<br>*Z*9*E*11-14:OAc<br>*Z*11-16:OAc<br>*Z*9-14:Ald	[4]
5	*Spodoptera dolichos* Fabricius	*Z*9-14:OAc<br>*Z*9*E*12-14:OAc	[1]
6	*Spodoptera eridania* Stoll	*Z*9-14:OAc<br>*Z*9*E*12-14:OAc<br>*Z*9*Z*12-14:OAc<br>*Z*9*E*11-14:OAc<br>*Z*11-16:OAc<br>*Z*9-14:OH	[5]
7	*Spodoptera evanida* Guenée	*Z*9-14:OAc<br>*Z*9*E*12-14:OAc	[1]
8	*Spodoptera exempta* Walker	*Z*9-14:OAc<br>*Z*9*E*12-14:OAc<br>*Z*9-14:Ald<br>*Z*9-14:OH<br>*Z*11-16:OAc<br>*Z*11-14:OAc	[6]
9	*Spodoptera exigua* Hübner	*Z*9*E*12-14:OAc<br>*Z*9-14:OAc<br>*Z*11-16:OAc<br>*Z*9*E*12-14:OH<br>*Z*9-14:OH<br>*Z*11-16:OH	[7]
10	*Spodoptera frugiperda* Smith	*Z*7-12:OAc<br>*E*7-12:OAc<br>12:OAc<br>*Z*9-12:OAc<br>*Z*9-14:OAc<br>*Z*10-14:OAc<br>14:OAc<br>Z11-16:OAc<br>*Z*11-14:OAc	[8]

11	*Spodoptera latifascia* Walker	*Z*9-12:OAc<br>*Z*11-14:OAc<br>*Z*9*E*12-14:OAc<br>*E*9*E*12-14:OAc<br>*Z*9*E*11-14:OAc<br>*Z*11-16:OAc<br>*Z*9-14:Ald	[4]
12	*Spodoptera littoralis* Boisduval	*Z*9*E*11-14:OAc<br>*Z*9-14:OAc<br>*E*11-14:OAc<br>14:OAc<br>*Z*11-14:OAc<br>*Z*9*E*12-14:OAc<br>*E*10*E*12-14:OAc	[9]
13	*Spodoptera litura* Fabricius	*Z*9*E*11-14:OAc<br>*Z*9*E*12-14:OAc<br>*Z*9-14:OAc<br>*E*11-14:OAc	[10]
14	*Spodoptera pectinicornis* Hampson	*Z*7-12:OAc	[11]
15	*Spodoptera praefica* Grote	*Z*7-12:OAc<br>*Z*7-12:OH<br>*Z*9-14:OAc<br>*Z*11-16:OAc	[12]
16	*Spodoptera sunia* Guenée	*Z*9-14:OAc<br>*Z*9*E*12-14:OAc<br>*Z*9-14:OH<br>*Z*11-16:OAc	[13]
17	*Spodoptera triturata* Walker	*Z*9-14:OAc<br>*Z*9*E*12-14:OAc<br>*E*9-14:OAc	[2]
18	*Spodoptera albula* Walker	*Z*9-14:OAc<br>*Z*9*E*12-14:OAc<br>*Z*9-14:OH<br>*Z*11-16:OAc	[14]

References

1 Lalanne Cassou, B., Silvain, J., Monti, L. & Malosse, C. Description of a new species of Spodoptera from French Guiana: *Spodoptera descoinsi* (Lepidoptera: Noctuidae: Amphipyrinae), discovered with sexual attractants. *Annales de la Societe Entomologique de France (France)* (1994).

2 Campion, D. Sex pheromones and their uses for control of insects of the genus Spodoptera in *Internationaal Symposium over Fytofarmacie en Fytiatrie* (1975).

3 Kurihara, M., Usui, K., Uchiumi, K. & Tatsuki, S. Sex-pheromone of the lawn grass cutworm moth, *Spodoptera depravata* (Butler) (Lepidoptera, Noctuidae). *Appl. Entomol. Zool.* **35**, 323-324 (1991).

4 Monti, L., Lalanne-Cassou, B., Lucas, P., Malosse, C. & Silvain, J.-F. Differences in sex pheromone communication systems of closely related species: *Spodoptera latifascia* (walker) and *S. descoinsi* lalannecassou & silvain (Lepidoptera: Noctuidae). *J. Chem. Ecol.* **21**, 641-660 (1995).

5 Teal, P., Mitchell, E., Tumlinson, J., Heath, R. & Sugie, H. Identification of volatile sex pheromone components released by the southern armyworm, *Spodoptera eridania* (Cramer). *J. Chem. Ecol.* **11**, 717-725 (1985).

6 Cork, A., Murlis, J. & Megenasa, T. Identification and field testing of additional components of female sex pheromone of African armyworm, *Spodoptera exempta* (Lepidoptera: Noctuidae). *J. Chem. Ecol.* **15**, 1349-1364 (1989).

7 Acín, P., Rosell, G., Guerrero, A. & Quero, C. Sex pheromone of the Spanish population of the beet armyworm *Spodoptera exigua*. *J. Chem. Ecol.* **36**, 778-786 (2010).

8 Unbehend, M. *et al.* Geographic variation in sexual attraction of Spodoptera frugiperda corn-and rice-strain males to pheromone lures. *PLOS ONE* **9**, e89255 (2014).

9 Munoz, L., Rosell, G., Quero, C. & Guerrero, A. Biosynthetic pathways of the pheromone of the Egyptian armyworm *Spodoptera littoralis*. *Physiol. Entomol.* **33**, 275-290 (2008).

10 Sun, F. & Hu, Y. The sex pheromone communication system of *Spodoptera litura* (Fabricius). *Acta. Entomol. Sin.* **45**, 404-407 (2001).

11 Van Hai, T., Son, P. K., Inomata, S.-I. & Ando, T. Sex attractants for moths of Vietnam: Field attraction by synthetic lures baited with known lepidopteran pheromones. *J. Chem. Ecol.* **28**, 1473-1481 (2002).

12 Landolt, P. J., Smithhisler, C., Adams, T. & Zack, R. S. An improved multi‐component sex attractant for trapping male western yellowstriped armyworm, *Spodoptera praefica* (Grote) (Lepidoptera: Noctuidae). *Agric. For. Entomol.* **5**, 333-339 (2003).

13 Bestmann, H., Attygalle, A., Schwarz, J., Vostrowsky, O. & Knauf, W. Identification of sex pheromone components of *Spodoptera sunia* Guenée (Lepidoptera: Noctuidae). *J. Chem. Ecol.* **14**, 683-690 (1988).

14 Meagher, R. L., Brambila, J. & Hung, E. Monitoring for exotic *Spodoptera* species (Lepidoptera: Noctuidae) in Florida. *Fla. Entomol.* **91**, 517-522 (2008).

Table S2: Polymerase Chain Reaction primers used in this study.

Forward (5'-3')	Reverse (5'-3')	Application
Os_F1_deg:<br>ACN GGH TTY MTD GGV AA	FAR_Gen_R:<br>GMTTTKGTGTANGYRTAYGTRTTHGG	Degenerate PCR
Os_F2_deg:<br>YAYRTDTCBACWGCHTA	FAR_Gen_R:<br>GMTTTKGTGTANGYRTAYGTRTTHGG	Degenerate PCR
FAR_LAT_F:<br>ACNGGNGSNACNGGNTT	FAR_Gen_R:<br>GMTTTKGTGTANGYRTAYGTRTTHGG	Degenerate PCR
Os_F1_deg:<br>ACN GGH TTY MTD GGV AA	Os_R2_deg:<br>RTADGCWGTVGAHAYRT	Degenerate PCR
Sex_FARI_RACE_F1:<br>ATGTCGCAGCCAATGTTCAGTTT	Sex_FARI_RACE_R1:<br>TAGGCTGTGGAAATATGGACAAATGC	RACE
Sex_FARI_RACE_F2:<br>GGCATTTGTCCATATTTCCACAG	Sex_FARI_RACE_R2:<br>AAACTGAACATTGGCTGCGACA	Nested RACE
SlitFARI_RACE_F1:<br>ATGTGGCAGCCAGTGTCCAGTTT	SlitFARI_RACE_R1:<br>TAGGCGGTGGACACATGGACAAAGGC	RACE
SlitFARI_RACE_F2:<br>GGCCTTTGTCCATGTGTCCACCG	SlitFARI_RACE_R2:<br>AAACTGGACACTGGCTGCCACA	Nested RACE
SexpgFARI FL_F:<br>TAAAATGACGTATAGACAAATAAATG	SexpgFARI FL_R:<br>TTAACTACGTTTCTTCATTAAGAACT	Functional gene expression
SlitpgFARI FL_F:<br>TAAAATGACGTATAGACAAATAAATG	SlitpgFARI_FL_Rev<br>TCT TTA AAT TAA TAA ATT ATG TAC	Functional gene expression
SlitpgFAR II_RACE_F<br>GTGGTCATTCACTCAGCAGCCACA	SlitpgFAR II_RACE_R<br>CCAGTTCGCCAGCCAGCCTC	RACE
SexpgFARII_RACE_F<br>GTGGTCATCCATTCAGCAGCAACT	SexpgFARII_RACE_R<br>CCAGTTCGCCAACCAGCCCC	RACE
SlitpgFARII_FL_F<br>ATGGTTGTGTTGACTTCGAA	SlitpgFARII_FL_R<br>TTA TTT TAT CTT TTC CAA AAA C	Functional gene expression
SexpgFARII_FL_F<br>ATGGTTGTGTTGACTTCGAA	SexpgFARII_FL_R<br>TTATTTTTTTTTTCCAAAAAC	Functional gene expression
SexpgFARIqrt_F:<br>TTCCTTCAGCAGCCACAGCA	SexpgFARIqrt_R:<br>CCTTCGCCGAGAAGCACTC	Quantitative PCR
SexpgFARIIqrt_F:<br>GAT CAG AGA GAA AAA GGG AC	SexpgFARIIqrt_R:<br>GTA TGC TGT CGA TAT GTG GAT G	Quantitative PCR
Sp_beta actin_F:<br>CCGTCCCCATCTACGAAGGTTACG	Sp_beta actin_R:<br>GCGGTGGCCATCTCCTGCTC	House-keeping gene/quantitative PCR
Bmori_pgFRA_F:<br>GAT CCA AGA TGT CAC ACA ATG GAA CTT TG	Bmori_pgFRA_R:<br>CTA TAA TTT ATT TTT GAA CAG ATG CTT GTT GA	Functional gene expression

Supporting information

Two *fatty acyl reductases* involved in moth pheromone biosynthesis

Antony et al.,

S1 Materials and Methods

Chemicals. Tetradecanoic acid methyl ester (C14:COOMe) and hexadecanoic acid Me (C16:COOMe) were purchased from Sigma (Dorset, England). Pentadecyl acetate (15:OAc), (*E*)-14-hexadecenoic acid Me (*E*14-16:COOMe), (*Z*)-9-tetradecenoic acid Me (*Z*9-14:COOMe), (*E*)-12-tetradecenoic acid Me (*E*12-14:COOMe), (*Z*)-12-tetradecenoic acid Me (*Z*12-14:COOMe), (*Z*)-9-hexadecenoic acid Me (*Z*9-16:COOMe), (*E*)-11-tetradecenoic acid Me (*E*11-14:COOMe), (*Z*)-11-tetradecenoic acid Me (*Z*11-14:COOMe), (*Z*)-11-hexadecenoic acid Me (*Z*11-16:COOMe), (*E*)-11-hexadecenoic acid Me (*E*11-16:COOMe), (*Z,Z*)-9,11-tetradecadienyl acid Me (*Z*9*Z*11-14:COOMe), (*Z,E*)-9,11-tetradecadienyl acid Me (*Z*9*E*11-14:COOMe), (*Z,E*)-9,12-tetradecadienyl acid Me (*Z*9*E*12-14:COOMe), (*E,E*)-10,12-tetradecadienyl acid Me (*E*10*E*12-14:COOMe), (*Z,Z*)-9,12-tetradecadienyl acid Me (*Z*9*Z*12-14:COOMe) and the corresponding fatty alcohols (used as authentic standards) were purchased from Pest Control of India Private Limited (Mumbai, India) and Pherobank (Netherlands). (*Z*)-7-dodecenyl acid Me (*Z*7-12:COOMe), (*Z*)-5-decenyl acid Me (*Z*5-10:COOMe), and the corresponding alcohols were kindly provided by the pheromone group of Lund University.

Functional Assay and GC-MS analysis. The functional assays were carried out in a yeast expression system following the procedures we previously described[15,16,17]. Briefly, the open reading frames of the *S. exigua*, *S. littoralis* and *B mori pgFAR* genes were cloned into the shuttle vector pYES2.1/V5-His TOPO (Invitrogen), and the resulting recombinant vectors were used to transform yeast of the INVSc1 strain of *S. cerevisae* (Invitrogen). When testing the conversion of individual precursors, aliquots of yeast cultures were suspended in induction medium containing 0.5 mM of the FAME methyl-ester precursor diluted in ethanol, and fatty alcohol products were extracted and analysed by gas chromatography-mass spectrometry under the conditions described below. When testing the alcohol production of yeast supplemented with precursor blends in the ratio as found in female pheromone glands[7,9] the total concentration of precursors was 0.5 mM and the culture medium was 5 ml, whereas all other parameters were kept unchanged.

Yeast cell extracts were subjected to GC-MS analysis on a Agilent 7850A GC coupled to a mass detector (Agilent 5975C) and equipped with a medium-polar INNOWax column (100% polyethylene glycol, 30 × 0.25 mm I.D., film thickness 0.25 mm, Agilent Technologies, USA). The GC-MS was operated in electron impact mode (70 eV), the injector was configured in split-less mode at 220°C, and helium was used as carrier gas (velocity: 30 cm/s). The oven temperature was set to 80°C for 1 min, then increased at a rate of 10°C/min up to 210°C, followed by a hold at 210°C for 15 min, and then increased at a rate of 10°C/min up to 230°C, followed by a hold at 230°C for 20 min.

15 Lassance, J. M. *et al.* Functional consequences of sequence variation in the pheromone biosynthetic gene pgFAR for Ostrinia moths. *Proc. Natl. Acad. Sci.USA* **110**, 3967-3972, doi:10.1073/pnas.1208706110 (2013).

16 Lassance, J.-M. *et al.* Allelic variation in a fatty-acyl reductase gene causes divergence in moth sex pheromones. *Nature* **466**, 486-489 (2010).

17 Liénard, M. A., Hagström, Å. K., Lassance, J.-M. & Löfstedt, C. Evolution of multicomponent pheromone signals in small ermine moths involves a single fatty-acyl reductase gene. *Proc. Natl. Acad. Sci. USA* **107**, 10955-10960 (2010).
